# Prevalence and incidence of sarcoidosis in Korea: a nationwide population-based study

**DOI:** 10.1186/s12931-018-0871-3

**Published:** 2018-08-28

**Authors:** Hee-Young Yoon, Hyeong Min Kim, Ye-Jee Kim, Jin Woo Song

**Affiliations:** 1 Department of Pulmonary and Critical Care Medicine, Asan Medical Center, University of Ulsan College of Medicine, 88 Olympic-Ro 43-Gil, Songpa-Gu, Seoul 05505 Republic of Korea; 2 Department of Clinical Epidemiology and Biostatistics, Asan Medical Center, University of Ulsan College of Medicine, 88 Olympic-Ro 43-Gil, Songpa-Gu, Seoul 05505 Republic of Korea

**Keywords:** Age distribution, Epidemiology, Sarcoidosis, Sex distribution, Population dynamics

## Abstract

**Background:**

The prevalence and incidence of sarcoidosis varies worldwide. We estimated the prevalence and incidence of sarcoidosis in Korea using nationwide claims data from the Korean Health Insurance Review and Assessment Service.

**Methods:**

Cases of sarcoidosis were identified for any visit between 2007 to 2016 that listed the *Korean Classification of Disease, 7th edition* code of sarcoidosis and rare incurable disease exempted calculation code. A narrow case definition was used as follows: 1) ≥ two sarcoidosis-coded visits within 1 year of the first claim, 2) no claims for other diseases that could form granuloma.

**Results:**

A total of 4791 patients (narrow, *n* = 2388) visited medical institutions for sarcoidosis during the study period; 2999 patients (narrow, *n* = 1696) were newly identified between 2009 and 2015. The sarcoidosis prevalence was 9.37 per 10^5^ people (narrow, 4.69) and was highest between ages 60–69 years. The incidence rate was 0.85 per 10^5^ population at risk (narrow, 0.48), with the highest incidence rate between ages 50–59 years. For incident cases (mean age: 48.5 year), the age distribution in whole population and females showed monophasic patterns peaking at aged 50–59 years, while males had biphasic incidence peak at aged 30–39 years and 60–69 years. The annual incidence rates showed increasing trends from 0.85 per 10^5^ population at risk in 2009 to 0.97 per 10^5^ population at risk in 2015.

**Conclusions:**

In comparison with previous reports, the prevalence and incidence of sarcoidosis in Korea have increased and middle-aged women showed the highest risk.

**Electronic supplementary material:**

The online version of this article (10.1186/s12931-018-0871-3) contains supplementary material, which is available to authorized users.

## Background

Sarcoidosis affects people of all ages, genders, and ethnicities, and its incidence and clinical manifestations vary widely throughout the world due to differences in environmental and genetic factors [1–5]. The highest incidence rates have been reported in Northern Europe (10–25 per 10^5^ population at risk) [6–8] and in African Americans (18–40 per 10^5^ population at risk) [9, 10]. The annual incidence rate in Japan is low (1–2 in 10^5^ population at risk) and appears to have a bimodal age distribution, with one peak at ages 25–34 years and another at ages 60–64 years [8, 11], while Western countries showed a single incident peak at 55–64 years [9]. Sarcoidosis usually occurs more frequently in females [8, 10–12], but male predominance has been observed in Northern Europe [7, 13].

In Korea, two nationwide surveys were conducted by the Korean Academy of Tuberculosis and Respiratory Disease (KATRD) in 1992 and 2000 [14, 15]. However, the survey was conducted by using questionnaires on biopsy-proven cases treated in hospitals with > 80 beds. Furthermore, the development of new diagnostic tools and increasing health screening may have resulted in significant epidemiological changes in recent years.

In Korea, nearly all of the population (> 50 million people) are covered by the National Health Insurance (NHI) system, which is a nationwide mandatory insurance scheme provided by the Korean government. The aim of this study was to use the NHI claims data to estimate the prevalence and incidence of sarcoidosis in Korea.

## Methods

### Data source

De-identified health claims data between January 1, 2007, and December 31, 2016, were extracted from the database of the Health Insurance and Review Agency (HIRA). These data were primarily based on the Korean NHI scheme, which covers 97% of the Korean population; claims from the remaining 3% of the population (who are covered by the Medical Assistance Program or the Medial Care for Patriots and Veterans Affairs Scheme) were also reviewed by the HIRA. Medical institutions transfer medical claims data electronically to the HIRA, which are then integrated into its claims database. The claims database provides healthcare utilization information for both inpatients and outpatients and includes patient demographics, diagnosis, diagnostic procedures, and prescribed medication. The *Korean Classification of Disease, 7th edition* (*KCD-7*), modification of the *International Classification of Disease and Related Health Problems, 10th revision* were used to code diagnoses (Additional file 1: Table S1). Data on demographics, other accompanying diagnostic codes, and diagnostic procedures were collected using the HIRA coding system. Prescription information included brand and generic name, prescription date, and duration.

In 2006, the NHI initiated a rare intractable diseases (RID) registration program, which includes 167 conditions, including sarcoidosis. Patients who met the diagnostic criteria with physician certification were offered up to 90% copayment reduction after RID program registration [16–19]. Following registration, both sarcoidosis-related *KCD-7* classification (D86) and RID code (V111) were listed to sarcoidosis-related claims. For more accurate diagnosis, RID code related to the sarcoidosis was used in this study. Because the NHI could refuse to pay hospital costs if diagnosis did not meet specific criteria, cases are reviewed by medical institutions prior to submission to the NHI and a reliable diagnosis can be presumed. There have been several prior studies on the incidence and prevalence of other rare incurable disease using the same RID registration database [16, 17, 19].

### Ethical approval

This study was approved by the Institutional Review Board of Asan Medical Center (2017–0627).

### Study population

People with sarcoidosis were identified in the claims data by a listing of the *KCD-7* code of sarcoidosis (D86.x) as a diagnosis and RID exempted calculation code (V111) in the secondary or tertiary care medical institutions in Korea (a broad case definition). To be registered in the RID program, patients were required to have involvement of specific organs (lung, skin, lymph node, heart, eye, nervous system, muscle, joint, and other organs) and imaging and pathologic findings compatible with sarcoidosis.

To complement the disadvantages of using claim data (overestimation or indeterminacy of diagnosis), and to ensure robust case definitions, a narrow case definition was also used for estimating prevalence and incidence and was defined as patients with 1) at least two sarcoidosis-coded visits within a year of the first sarcoidosis-related claim and 2) no claims for any other causes of granuloma formation, including mycobacterium (A15–19, A319), mycoses (B35–49), and malignant neoplasm (C00–97) within 90 days before or after the first claim.

### Statistical analysis

Patients identified with sarcoidosis during the study period were included in the prevalence estimates. Based on prevalent cases for 2007 and 2008, newly diagnosed patients from 2009 to 2016 were added cumulatively every year; the prevalence was estimated as the number of all patients identified with sarcoidosis divided by the total population for the year of 2016. The total population was obtained from the Korean Statistical Information Service (http://kosis.kr). For the estimate of incidence, the date of the earliest claim of sarcoidosis was defined as the index date and was considered as the incident time, and the patient was considered to be an incident case in that year. To remove any potential pre-existing cases of sarcoidosis, a clearance period was established by excluding cases identified in the first 2 years (2007–2008). The annual incidence rate from 2009 to 2015 was calculated as the number of newly identified cases in a corresponding year divided by the population at risk. Cases identified in 2016 were excluded due to insufficient 1 year follow-up data. The population at risk for the incidence estimates was determined by removing identified pre-existing cases of sarcoidosis from the mid-year population. To compare incidence rates over time, standardized incidence for the Korean population in 2015 served as reference, using direct standardization. The 95% confidence interval (CI) of the prevalence and incidence rates was estimated using a Poisson distribution. In the incidence cases, age, gender, other accompanying diagnostic codes (comorbidities), and codes for medication and diagnostic tests were analyzed. Medication use for sarcoidosis was identified in the prescription record within a year of the index date. All statistical analyses were performed using SAS Enterprise Guide software (version 6.1, SAS Institute, Inc., Cary, NC).

## Results

### Prevalence

**Fig. 1:**
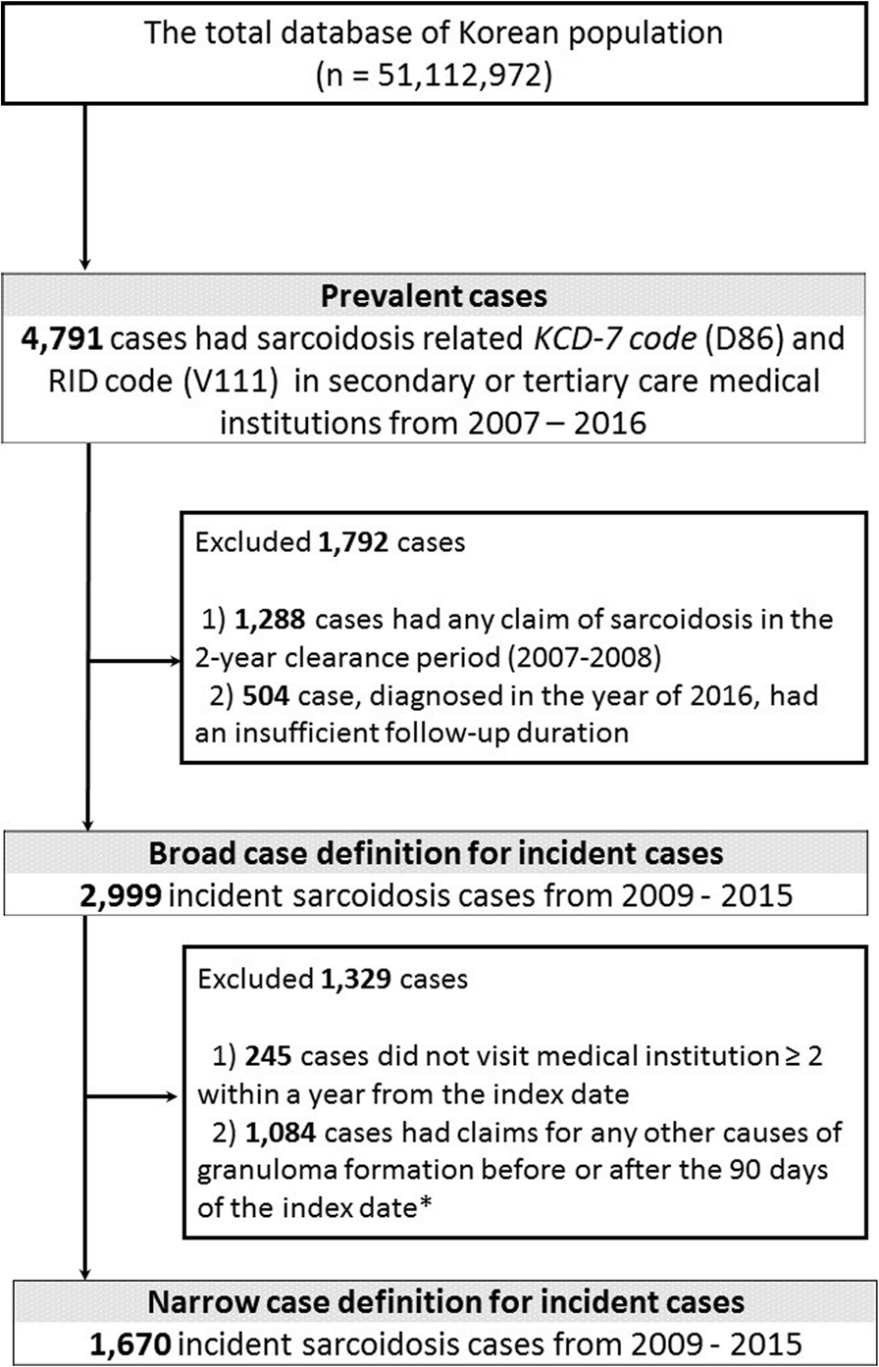
Identification of sarcoidosis cases from the Health Insurance Review and Assessment Service database. *Index date: the date of the earliest claim of sarcoidosis

A total of 4791 patients (male: *n* = 1864, female: *n* = 2927) had at least one visit with a sarcoidosis-related *KCD-7* and RID code during the study period (Fig. 1). The 10 year prevalence was 9.37 per 10^5^ people (95% CI: 9.11–9.64), with prevalence in females and males being 11.44 (95% CI: 11.03–11.86) and 7.30 (95% CI: 6.97–7.64), respectively (Additional file 1: Table S2). The mean age of prevalent cases was 53.2 ± 13.9 years (males: 48.9 ± 15.0 years, females: 56.1 ± 12.3 years), and the female to male ratio was 1.57. By the narrow definition, 2388 patients (males: *n* = 949, females: *n* = 1439) were identified and the 10 year prevalence was 4.69 (95% CI: 4.50–4.88) per 10^5^ people (males: 3.73 [95% CI: 3.49–3.97] per 10^5^ people, females: 5.64 [95% CI: 5.36–5.94] per 10^5^ people).

**Fig. 2:**
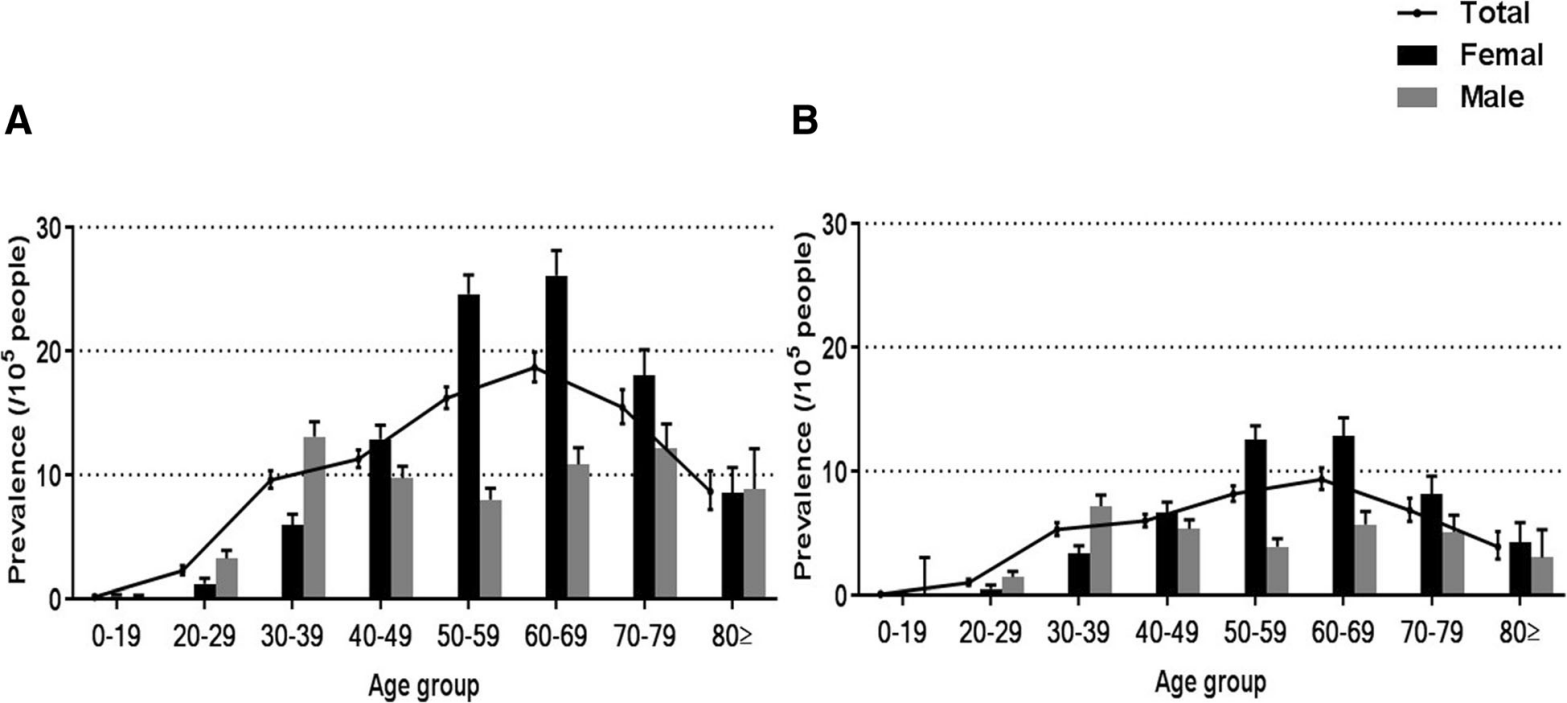
The prevalence of sarcoidosis stratified by age and sex. **a**. Prevalence data of the whole group from 2007 to 2016. **b**. Prevalence according to the narrow definition from 2007 to 2015. Data are presented as the mean ± 95% confidence interval

The peak prevalence of the total population was 60–69 years (Additional file 1: Table S2). When stratified by sex, the highest prevalence in females was seen in those aged 60–69 years; the prevalence in males showed bimodal peaks, with one at 30–39 years and another at 70–79 years (Fig. 2a). When the narrow definition was used, the age-specific prevalence showed a similar pattern, although the second peak age in males was 60–69 years (Fig. 2b).

### Incidence

**Fig. 3:**
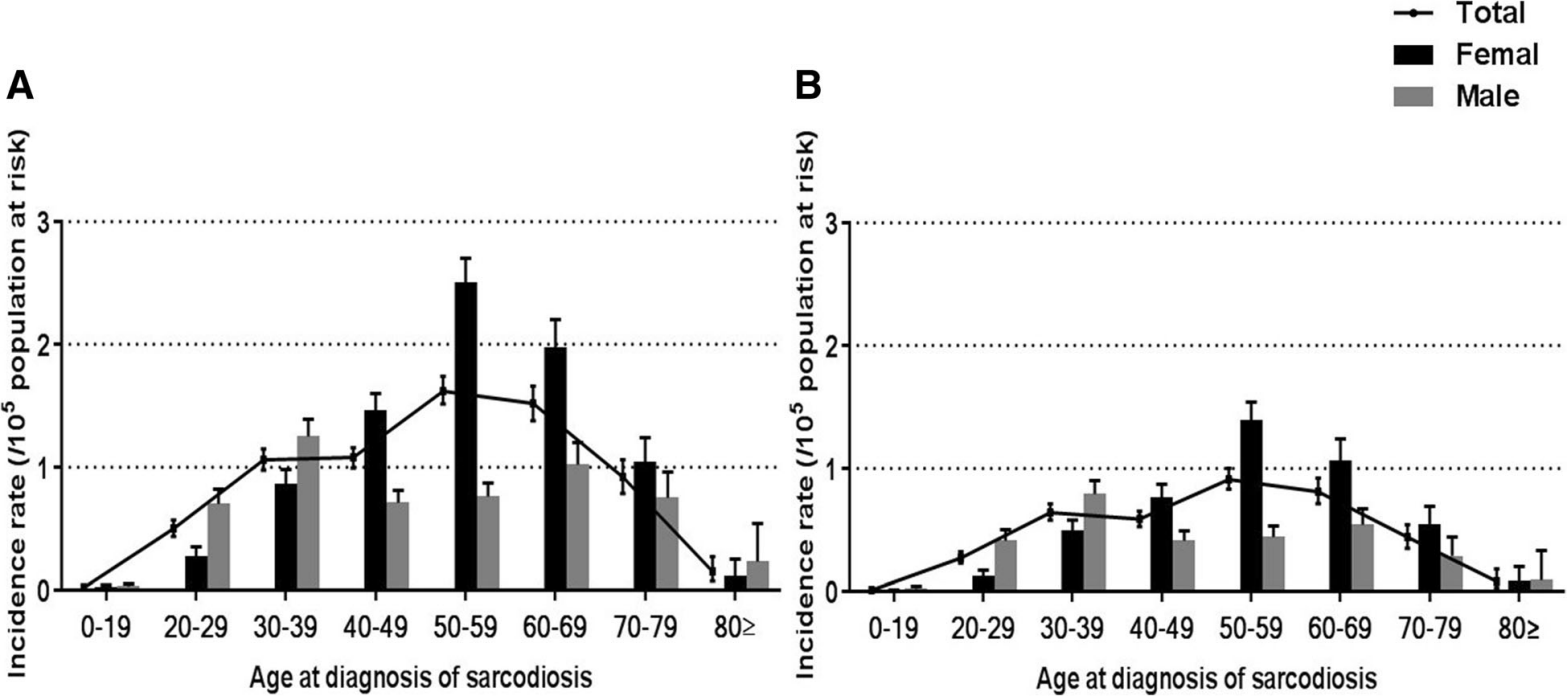
The incidence of sarcoidosis stratified by age and sex. **a**. Incidence data of the whole group from 2009 to 2015. **b**. Incidence rate by the narrow definition from 2009 to 2015. Data are presented as the mean ± 95% confidence interval

A total of 2999 incident cases (males: *n* = 1176, females: *n* = 1823) were identified from 2009 to 2015 (Fig. 1). The incidence rate was 0.85 (95% CI: 0.82–0.88) per 10^5^ population at risk and was higher in females (1.04 [95% CI: 0.99–1.08] per 10^5^ population at risk) than males (0.67 [95% CI: 0.63–0.71] per 10^5^ population at risk). The age-specific incidence rates in the whole group and females peaked at the age of 50–59 years, while that in males exhibited bimodal peaks at 30–39 years and 60–69 years (Fig. 3a; Additional file 1: Table S3). The female-to-male ratio among the incident cases was 1.55, and the average age at registration was 48.5 ± 13.5 years (males: 44.0 ± 14.6 years, females: 51.4 ± 12.0 years).

By the narrow definition, 1670 incident cases (males: *n* = 686, females: *n* = 984) were identified (Fig. 1). The incidence rate was 0.47 (95% CI: 0.45–0.50) per 10^5^ population at risk during the study period (males: 0.39 [95% CI: 0.36–0.42] per 10^5^ population at risk, females: 0.56 [95% CI: 0.52–0.60] per 10^5^ population at risk). Age-specific incidence rates of the narrow definition also showed trends similar to those of the broad definition (Fig. 3b).

**Fig. 4:**
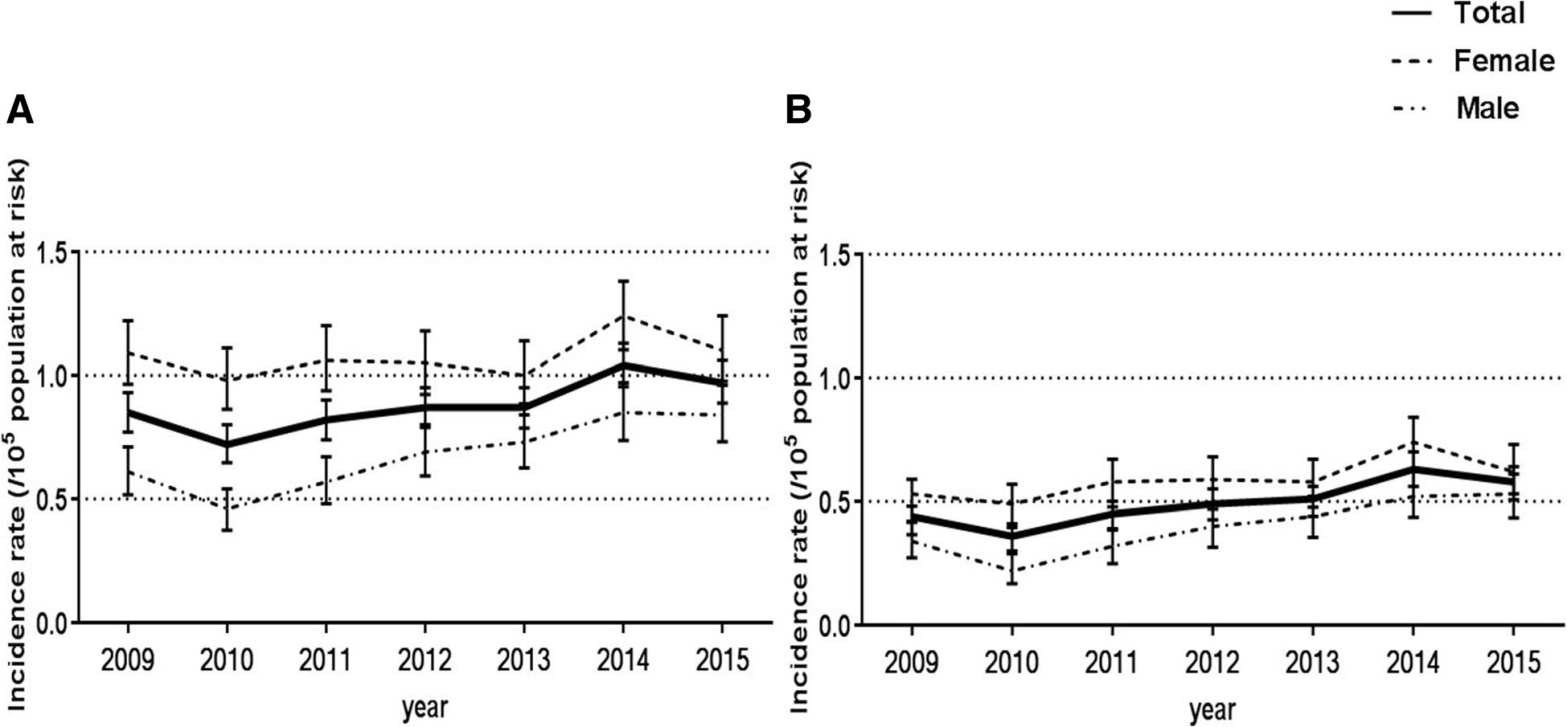
Age- and sex- standardized annual sarcoidosis incidence. **a**. Incidence data of the whole group from 2009 to 2015. **b**. Incidence rate according to the narrow definition from 2009 to 2015. Data are presented as the mean ± 95% confidence interval

The age- and sex-standardized annual incidence rates showed increasing trends from 0.85 (95% CI: 0.77–0.93) per 10^5^ population at risk in 2009 to 0.97 (95% CI: 0.89–1.06) per 10^5^ population at risk in 2015 (Fig. 4a). The incidence rate in males increased from 0.61 (95% CI: 0.52–0.71) per 10^5^ population at risk in 2009 to 0.84 (95% CI: 0.73–0.96) per 10^5^ population at risk in 2015; however, the incidence in females remained constant (1.09 [95% CI: 0.96–1.22] in 2009, 1.10 [95% CI: 0.981.24] per 10^5^ population at risk in 2015). By the narrow definition, the age- and sex-standardized annual incidence rates also increased from 0.42 (95% CI: 0.37–0.48) to 0.57 (95% CI: 0.51–0.64) per 10^5^ population at risk between 2009 and 2015 (males: 0.34 [95% CI: 0.27–0.42] to 0.52 [95% CI: 0.43–0.61] per 10^5^ population at risk, females: 0.50 [95% CI: 0.42–0.59] to 0.62 [95% CI: 0.53–0.73] per 10^5^ population at risk; Fig. 4b). The incidence rate tended to increase in the second and third quarters compared to the first and fourth quarters, and this tendency was particularly pronounced in females (Additional file 2: Figure S1).

### Comorbidities among incident cases

Of the 2999 incident cases, 32.0% had an accompanying diagnosis code including erythema nodosum (L52, 1.2%), heart failure (I50, 5.9%), and Bell’s palsy (G51.0, 1.6%), respectively (Additional file 1: Table S1 and S4). Respectively, 8.3% and 0.3% of incident cases had codes of uveitis (H20.0, H22.0) and multiple cranial nerve palsies in sarcoidosis (G53.2).

### Diagnostic methods and medication in incident cases

Diagnostic bronchoscopy was conducted in 50.6% of incident cases before or after 3 months of the first claim (Table 1). The proportion of patients with claims for bronchoscopy increased from 44.0% in 2009 to 56.4% in 2015 (Additional file 2: Figure S2). A total of 935 (51.3%) incident cases had claims for biopsy including surgical lung biopsy (19.1%) and skin biopsy (13.1%).

**Table 1:** Diagnostic methods of sarcoidosis

Diagnostic methods	N (%)
Bronchoscopy	1518 (50.6)
BAL	892 (29.7)
TBLB	504 (16.8)
Surgical lung biopsy	573 (19.1)
Skin biopsy	392 (13.1)
Musculoskeletal biopsy	45 (1.5)
Others^a^	293 (9.8)
All subjects undergoing biopsy	935 (51.3)
Unknown	796 (26.5)

*BAL* bronchoalveolar lavage, *TBLB* transbronchial lung biopsy

^a^Others included needle, incisional, and surgical biopsy of the liver, the heart, and organs other than the lung, skin, muscle, bone, and joints

The data showed claims for systemic steroids at least once within a year of the first claim in 78% of incident cases (Table 2). Of these, 1527 (50.9%) received systemic steroid for > 30 days (initial 30 day mean prescribed doses: 33.0 ± 25.7 mg based on prednisolone equivalents). Topical steroid (20.9%), other immunosuppressant (8.8%), and hydroxychloroquine (4.1%) were also claimed within a year of the earliest sarcoidosis claim. Most medication was initiated within 4 months of the index date, with the exception of topical steroid.

**Table 2:** Medication use in cases of incident sarcoidosis

	N (%)	Mean time from diagnosis to first treatment (days)
Systemic steroid	2340 (78.0)	49.0 (85.9)
prednisolone	1666 (55.6)	44.7 (81.9)
methylprednisolone	885 (29.5)	106.9 (113.8)
dexamethasone	659 (22.0)	153.5 (110.2)
use for > 30 days	1527 (50.9)	19.4 (45.6)
Topical steroid	627 (20.9)	155.5 (114.7)
hydrocortisone	180 (6.0)	172.2 (110.6)
betamethasone	159 (5.3)	172.0 (112.6)
methylprednisolone	148 (4.9)	125.9 (120.3)
Other immunosuppressant	263 (8.8)	96.5 (109.7)
methotrexate	131 (4.4)	91.7 (104.2)
cyclosporin	85 (2.8)	130.2 (117.3)
azathioprine	72 (2.4)	99.1 (112.0)
use for > 30 days	180 (6.0)	69.5 (85.9)
Hydroxychloroquine	123 (4.1)	50.3 (86.6)
use for > 30 days	97 (3.2)	47.3 (80.6)
None	575 (19.2)	

Data are presented as number (%) or mean (standard deviation), unless otherwise indicated

## Discussion

This is the first population-based study of the prevalence and incidence of sarcoidosis in Korea to use data from a nationwide medical claims database. Compared to previous reports, the prevalence and incidence of sarcoidosis in Korea have increased. The age of diagnosis was also markedly increased, and middle-aged women had the highest risk.

The KATRD study group estimated the incidence of sarcoidosis based on biopsy-proven cases diagnosed between 1980 and 1992 in 213 hospitals with more than 100 beds. According to these results, there were 133 confirmed sarcoidosis cases and the peak onset age was 30–39 years [14]. The study by Kim et al. also reported 309 patients with sarcoidosis identified in hospitals with over 80 beds [15]. The incidence in 1998 was 0.13 per 10^5^ population and peaked in both males and females in their thirties. In the present study, the female predominance observed in previous studies was confirmed, but the peak ages were higher. The recent studies on epidemiology of sarcoidosis in other countries also support our findings [7, 20–22]. In previous studies in Sweden and Japan, the mean age of sarcoidosis at the tme of diagnosis was 56 and 54 years, respectively [7, 22]. As Korean population has been gradually aging for decades, (the proportion of people over 65: 7.2% [2000] vs .13.8% [2017]; Statistics Korea, http://kostat.go.kr), the increase in the age of diagnosis in patients with sarcoidosis may be due to the aging population. Population-based studies in Sweden and Japan have also suggested population aging as the cause of increased diagnosis age of sarcoidosis [7, 22]. Since older sarcoidosis patients have a lower spontaneous resolution of chest x-ray than younger patients [23], the detection rate for older patients might be increased. Because Korea is a racially homogeneous country, environmental factors may be another cause for increased age at diagnosis. As industrialization progresses, there is a greater chance of encountering substances that generate granulomatous immunological reactions, including metal, inorganic, and organic dust [2–5, 24]. Accumulation of exposure to substances over time may be associated with the higher incidence of sarcoidosis in higher age group.

Compared with previous studies conducted by nationwide surveys in 1992 and 2000 [14, 15], we found that the incidence and prevalence of sarcoidosis have increased during the study period. The present study also showed a gradual increase in incidence rates over time. Similar to our results, Park et al. reported that incidence of sarcoidosis increased 1.6 times over from 2003 to 2015 [25]. This observation may, in part, be due to higher detection rates as a result of the development of new diagnostic tools, such as bronchoscopy and endobronchial ultrasound (EBUS). In a Korean single center study, more than 90% of patients with sarcoidosis between 1996 and 2014 were diagnosed after the introduction of EBUS bronchoscopy in 2008 [26]. In Korea, EBUS bronchoscopy was first introduced in 2005 and its use has become widespread. Although this study was unable to collect data on EBUS, which is not reimbursed, it did show an increasing trend for bronchoscopy since 2007, suggesting that the proportion of cases diagnosed by EBUS bronchoscopy would have increased. It is also possible that detection has increased since the RID program, which was established in 2006, increased patients’ access to medical facilities. Another possible cause is that an increase in the rate of health screening for Korean conducted by the National Health Insurance Service (NHIS) may affect the incidence of sarcoidosis. The NHIS reported that the health screening rate has steadily increased from 60.0% in 2007 to 77.7% in 2016 (https://www.nhis.or.kr). Because the health screening program includes a chest x-ray and blood tests, increased health screening can increase the chance of detecting asymptomatic patients.

The age distribution of subjects with sarcoidosis in our study was similar to that of subjects in Western studies [3, 27, 28], which show a monophasic pattern, and was unlike that of Japanese and Scandinavian subjects, which show a biphasic distribution [11, 29]. On the other hand, the age distribution of males in the present study showed a bimodal pattern due to the presence of a younger peak in the age group 30–39 years that was distinct from that seen in Western populations with a single incidence peak in aged 40–59 years [3, 12, 30]. In a previous Korean study, the peak age of sarcoidosis in males was in their thirties [15], but the present study confirmed the second peak between ages 60–69 years. These changes in age distribution are similar to those observed in Japan in the 2000s [20].

Almost 70% of incident cases in the present study received one or more treatments within a year of the index date. Compared with other studies that showed a < 50% rate of steroid treatment for sarcoidosis [9, 27, 31, 32], the present study showed a higher rate of treatment. Because cases were restricted to those who visited secondary or tertiary care medical institutions and were enrolled in the RID program, this study may therefore include more severe patients who required treatment. As sarcoidosis shows a chronic clinical course and usually requires at least 3 months of treatment [33–36], we also limited treatment cases to those with > 30 days’ treatment-related claims to ensure that our data were robust. As a result, the rate of treated cases was 50.9%, similar to that seen in other studies [9, 27, 31, 32].

This study has the limitations. First, we identified sarcoidosis cases using diagnostic codes from a national insurance claims database; the diagnosis of these cases was not confirmed and may have resulted in an overestimation of prevalence and incidence rates. Therefore, we used the RID registration system, which requires the subject to meet the universal diagnostic criteria for registration, and cases were reviewed by medical institutions prior to submission to the NHI. We also used the narrow definition of sarcoidosis to estimate incidence and prevalence more accurately. Secondly, data were not available for procedures that are not reimbursed, such as EBUS. However, because most patients tended to undergo bronchoscopy screening prior to EBUS, we assumed that the claim data for bronchoscopy would reflect the status of claims for EBUS and found an increasing trend in the proportion of patients who underwent bronchoscopy.

## Conclusion

In conclusion, our data showed that, while the incidence and prevalence of sarcoidosis in Korea has increased compared with prior Korean studies, it remains lower than that documented in Western countries. We also observed a secular increase in sarcoidosis incidence which may be due to increased detection or improved recognition.

## Additional files

Additional file 1: **Table S1.** Matching diagnostic codes between the KCD-7 and ICD-10 classification. KCD-7, Korean Classification of Disease, 7th revsion; ICD-10, International Classification of Disease, 10th revision. **Table S2.** Number of patients with sarcoidosis and prevalence rate (per 10^5^ population) of sarcoidosis in Korea from 2007 to 2016. N, number of sarcoidosis. P, prevalence. CI, confidence interval. **Table S3.** Number of patients with sarcoidosis and incidence rate (per 10^5^ population at risk) of sarcoidosis in Korea from 2009 to 2015. N, number of sarcoidosis. I, Incidence. CI, confidence interval. *population at risk was defined by removing prevalent sarcoidosis cases from the mid-year total Korean population. **Table S4.** Accompanying diagnostic codes in incident sarcoidosis cases after registration. Data are presented as N (%), unless otherwise indicated. (DOCX 37 kb)

Additional file 2: **Figure S1.** A seasonal variation in sarcoidosis incidence rate from 2009 to 2015. Q1, the first quarter; Q2, the second quarter; Q3, the third quarter; Q4, the fourth quarter. **Figure S2.** Annual change in the proportion of incident cases with claims for bronchoscopy. (ZIP 82 kb)

## Abbreviations

CI: Confidence interval
EBUS: Endobronchial ultrasound
HIRA: Health insurance and review agency
KCD-7: The Korean Classification of Disease, 7th edition
NHI: The National Health Insurance
RID: Rare intractable diseases
