# Supplementary material for: Prevalence and incidence of sarcoidosis in Korea: a nationwide population-based study
Source: Respir Res. 2018 Aug 28;19:158. doi: 10.1186/s12931-018-0871-3 (PMC6114796; doi:10.1186/s12931-018-0871-3)
Supplement: Supplementary file 1 — Table S1. Matching diagnostic codes between the KCD-7 and ICD-10 classification. KCD-7, Korean Classification of Disease, 7th revsion; ICD-10, International Classification of Disease, 10th revision. Table S2. Number of patients with sarcoidosis and prevalence rate (per 105 population) of sarcoidosis in Korea from 2007 to 2016. N, number of sarcoidosis. P, prevalence. CI, confidence interval. Table S3. Number of patients with sarcoidosis and incidence rate (per 105 population at risk) of sarcoidosis in Korea from 2009 to 2015. N, number of sarcoidosis. I, Incidence. CI, confidence interval. *population at risk was defined by removing prevalent sarcoidosis cases from the mid-year total Korean population. Table S4. Accompanying diagnostic codes in incident sarcoidosis cases after registration. Data are presented as N (%), unless otherwise indicated. (DOCX 37 kb) [file 12931_2018_871_MOESM1_ESM.docx]

**Table S1** Matching diagnostic codes between the *KCD-7* and *ICD-10* classification

| KCD-7 | ICD-10 |
| --- | --- |
| D86 Sarcoidosis | D86 Sarcoidosis |
| D86.0 Sarcoidosis of lung | D86.0 Sarcoidosis of lung |
| D86.1 Sarcoidosis of lymph nodes | D86.1 Sarcoidosis of lymph nodes |
| D86.2 Sarcoidosis of lung with sarcoidosis of lymph nodes | D86.2 Sarcoidosis of lung with sarcoidosis of lymph nodes |
| D86.3 Sarcoidosis of skin | D86.3 Sarcoidosis of skin |
| D86.8 Sarcoidosis of other sites | D86.8 Sarcoidosis of other sites |
|  | D86.81 Sarcoid meningitis |
|  | D86.82 Multiple cranial nerve palsies in sarcoidosis |
|  | D86.83 Sarcoid iridocyclitis |
|  | D86.84 Sarcoid pyelonephritis |
|  | D86.85 Sarcoid myocarditis |
|  | D86.86 Sarcoid arthropathy |
|  | D86.87 Sarcoid myositis |
|  | D86.89 Sarcoidosis of other sites |
| D86.9 Sarcoidosis, unspecific | D86.9 Sarcoidosis, unspecific |
| E10-E14 Diabetes mellitus | E10-E14 Diabetes mellitus |
| E78 Disorders of lipoprotein metabolism and other lipidaemias | E78 Disorders of lipoprotein metabolism and other lipidaemias |
| G51.0 Bell’s palsy | G51.0 Bell palsy |
| G53.2 Multiple cranial nerve palsies in sarcoidosis | G53.2 Multiple cranial nerve palsies in sarcoidosis |
| H20.0 Acute and subacute iridocyclitis | H20.0 Acute and subacute iridocyclitis |
| H22.0 Acute and subacute iridocyclitis in infectious and parasitic diseases classified elsewhere iridocyclitis | H22.0 Acute and subacute iridocyclitis in infectious and parasitic diseases classified elsewhere iridocyclitis |
| I10-I15 Hypertensive diseases | I10-I15 Hypertensive diseases |
| I47 Paroxysmal tachycardia | I47 Paroxysmal tachycardia |
| I48 Atrial fibrillation and flutter | I48 Atrial fibrillation and flutter |
| I49 Other cardiac arrhythmias | I49 Other cardiac arrhythmias |
| I50 Hear failure | I50 Hear failure |
| I88 Nonspecific lymphadenitis | I88 Nonspecific lymphadenitis |
| I89 Other noninfective disorders of lymphatic vessels and lymph nodes | I89 Other noninfective disorders of lymphatic vessels and lymph nodes |
| J84 Other interstitial pulmonary diseases | J84 Other interstitial pulmonary diseases |
| L52 Erythema nodosum | L52 Erythema nodosum |
| M63.3 Myositis in sarcoidosis | M63.3 Myositis in sarcoidosis |
| N17-N19 Renal failure | N17-N19 Renal failure |

*KCD-7*, *Korean Classification of Disease, 7^th^ revision*; *ICD-10*, *International Classification of Disease, 10^th^ revision*.

**Table S2.** Number of patients with sarcoidosis and prevalence rate (per 10^5^ population) of sarcoidosis in Korea from 2007–2016

| Age group | Total | | | | Female | | | | Male | | | | Female-to-male ratio |
| --- | --- | --- | --- | --- | --- | --- | --- | --- | --- | --- | --- | --- | --- |
|  | N | Total Population | P | 95% CI | N | Total Population | P | 95% CI | N | Total Population | P | 95% CI |  |
| 0–19 years | 17 | 10,167,571 | 0.17 | 0.10-0.27 | 9 | 4,903,314 | 0.18 | 0.08-0.35 | 8 | 5,264,257 | 0.15 | 0.07-0.30 | 1.21 |
| 20–29 years | 152 | 6,695,952 | 2.27 | 1.92-2.66 | 38 | 3,169,220 | 1.20 | 0.85-1.65 | 114 | 3,526,732 | 3.23 | 2.67-3.88 | 0.37 |
| 30–39 years | 724 | 7,538,707 | 9.60 | 8.92-10.33 | 220 | 3,688,221 | 5.96 | 5.20-6.81 | 504 | 3,850,486 | 13.09 | 11.97-14.28 | 0.46 |
| 40–49 years | 984 | 8,716,227 | 11.29 | 10.59-12.02 | 554 | 4,297,638 | 12.89 | 11.84-14.01 | 430 | 4,418,589 | 9.73 | 8.83-10.70 | 1.32 |
| 50–59 years | 1,338 | 8,247,924 | 16.22 | 15.36-17.12 | 1,006 | 4,092,335 | 24.58 | 23.09-26.15 | 332 | 4,155,589 | 7.99 | 7.15-8.90 | 3.08 |
| 60–69 years | 962 | 5,151,264 | 18.68 | 17.51-19.89 | 691 | 2,648,485 | 26.09 | 24.18-20.08 | 271 | 2,502,779 | 10.83 | 9.58-12.20 | 2.41 |
| 70–79 years | 491 | 3,173,638 | 15.47 | 14.13-16.90 | 325 | 1,804,079 | 18.01 | 16.11-10.59 | 166 | 1,369,560 | 12.12 | 10.35-14.11 | 1.49 |
| ≥ 80 years | 123 | 1,421,690 | 8.65 | 7.19-10.32 | 84 | 981,867 | 8.56 | 6.82-10.59 | 39 | 439,823 | 8.87 | 6.31-12.12 | 0.96 |
| Total | 4,791 | 51,112,972 | 9.37 | 9.11-9.64 | 2,927 | 25,585,157 | 11.44 | 11.03-11.89 | 1,864 | 25,527,815 | 7.30 | 6.97-7.64 | 1.57 |

N, number of sarcoidosis. P, prevalence. CI, confidence interval.

**Table S3.** Number of patients with sarcoidosis and incidence rate (per 10^5^ population at risk) of sarcoidosis in Korea from 2009–2015

| Age group | Total | | | | Female | | | | Male | | | | Female-to-male ratio |
| --- | --- | --- | --- | --- | --- | --- | --- | --- | --- | --- | --- | --- | --- |
|  | N | Population  at risk* | I | 95% CI | N | Population  at risk* | I | 95% CI | N | Population  at risk* | I | 95% CI |  |
| 0–19 years | 18 | 78,248,109 | 0.02 | 0.01–0.04 | 7 | 37,402,475 | 0.02 | 0.01–0.04 | 11 | 40,845,634 | 0.03 | 0.01-0.05 | 0.69 |
| 20–29 years | 235 | 47,256,633 | 0.50 | 0.44–0.57 | 62 | 22,622,442 | 0.27 | 0.21–0.35 | 173 | 24,634,191 | 0.70 | 0.60–0.82 | 0.39 |
| 30–39 years | 600 | 56,645,328 | 1.06 | 0.98–1.15 | 239 | 27,767,028 | 0.86 | 0.76–0.98 | 361 | 28,878,300 | 1.25 | 1.12–1.39 | 0.69 |
| 40–49 years | 658 | 61,185,356 | 1.08 | 0.99–1.16 | 438 | 30,068,409 | 1.46 | 1.32–1.60 | 220 | 31,116,947 | 0.71 | 0.62–0.81 | 2.06 |
| 50–59 years | 840 | 51,695,143 | 1.62 | 1.52–1.74 | 643 | 25,760,337 | 2.50 | 2.31–2.70 | 197 | 25,934,806 | 0.76 | 0.66–0.87 | 3.29 |
| 60–69 years | 457 | 30,162,447 | 1.52 | 1.38–1.66 | 309 | 15,680,785 | 1.97 | 1.76–2.20 | 148 | 14,481,663 | 1.02 | 0.86–1.20 | 1.93 |
| 70–79 years | 180 | 19,664,847 | 0.92 | 0.79–1.06 | 119 | 11,494,307 | 1.04 | 0.86–1.24 | 61 | 8,170,541 | 0.75 | 0.57–0.96 | 1.39 |
| ≥ 80 years | 11 | 7,392,026 | 0.15 | 0.07–0.27 | 6 | 5,222,142 | 0.11 | 0.04–0.25 | 5 | 2,169,884 | 0.23 | 0.07–0.254 | 0.50 |
| Total | 2,999 | 352,249,887 | 0.85 | 0.82–0.88 | 1,823 | 176,017,924 | 1.04 | 0.99–1.08 | 1,176 | 176,231,964 | 0.67 | 0.63–0.71 | 1.55 |

N, number of sarcoidosis. I, incidence. CI, confidence interval.

* The population at risk was defined by removing prevalent sarcoidosis cases from the mid-year Korean population

**Table S4.** Accompanying diagnostic codes in incident sarcoidosis cases after registration

|  | Total | Female | Male |
| --- | --- | --- | --- |
| Dyslipidemia (E78) | 1,780 (59.4) | 1,179 (64.7) | 601 (51.1) |
| Hypertension (I10-15) | 1,148 (38.3) | 736 (40.4) | 412 (35.0) |
| Diabetes mellitus (E10-14) | 990 (33.0) | 626 (34.3) | 364 (31.0) |
| Ischemic heart disease (I20-25) | 455 (15.2) | 281 (15.4) | 174 (14.8) |
| Arrhythmias (I47-49) | 276 (9.2) | 176 (9.7) | 100 (8.5) |
| Uveitis (H20.0, H22.0) | 247 (8.3) | 172 (9.4) | 75 (6.4) |
| Heart failure (I50) | 177 (5.9) | 107 (5.9) | 70 (6.0) |
| Interstitial pulmonary diseases (J84) | 173 (5.8) | 93 (5.1) | 80 (6.8) |
| Renal failure (N17-19) | 147 (4.9) | 76 (4.2) | 71 (6.0) |
| Bell’s palsy (G51.0) | 47 (1.6) | 34 (1.9) | 13 (1.1) |
| Erythema nodosum (L52) | 36 (1.2) | 29 (1.6) | 7 (0.6) |
| Multiple cranial nerve palsies in sarcoidosis (G53.2) | 10 (0.3) | 6 (0.3) | 4 (0.3) |
| Myositis in sarcoidosis (M63.3) | 6 (0.2) | 5 (0.3) | 1 (0.1) |

Data are presented as N (%), unless otherwise indicated.
