# Supplementary figures and images for: Prevalence and incidence of sarcoidosis in Korea: a nationwide population-based study
Source: Respir Res. 2018 Aug 28;19:158. doi: 10.1186/s12931-018-0871-3 (PMC6114796; doi:10.1186/s12931-018-0871-3)

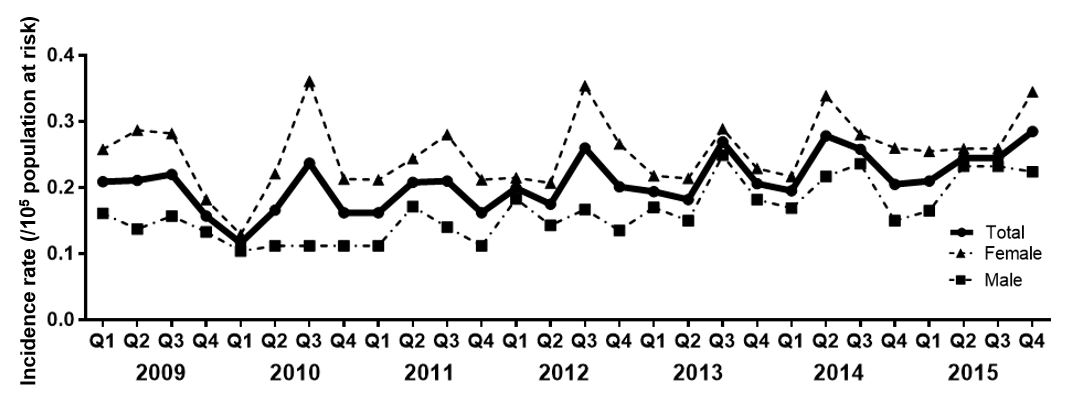

Supplement: Supplementary file 2 — Figure S1. A seasonal variation in sarcoidosis incidence rate from 2009 to 2015. Q1, the first quarter; Q2, the second quarter; Q3, the third quarter; Q4, the fourth quarter. Figure S2. Annual change in the proportion of incident cases with claims for bronchoscopy. (ZIP 82 kb) [file 12931_2018_871_MOESM2_ESM.zip › FigS1.JPG]

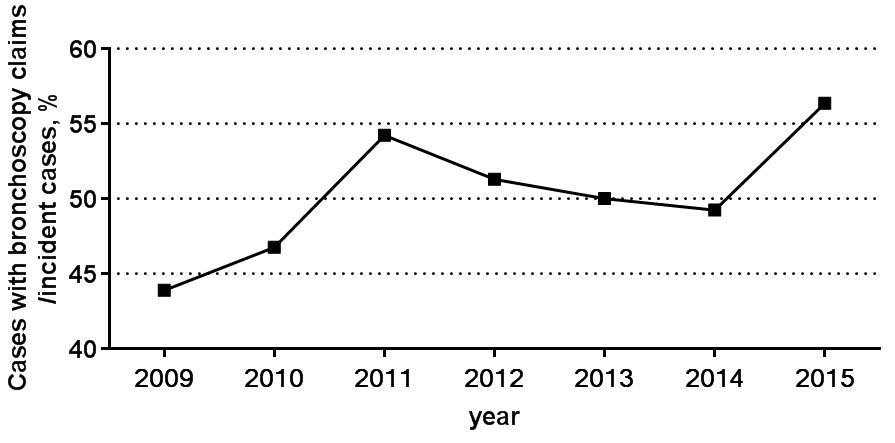

Supplement: Supplementary file 2 — Figure S1. A seasonal variation in sarcoidosis incidence rate from 2009 to 2015. Q1, the first quarter; Q2, the second quarter; Q3, the third quarter; Q4, the fourth quarter. Figure S2. Annual change in the proportion of incident cases with claims for bronchoscopy. (ZIP 82 kb) [file 12931_2018_871_MOESM2_ESM.zip › FigS2.JPG]
